# Production of Intestinal Mucins, sIgA, and Metallothionein after Administration of Zinc and Infection of *Ascaridia galli* in Chickens: Preliminary Data

**DOI:** 10.3390/life13010067

**Published:** 2022-12-26

**Authors:** Martin Levkut, Mária Levkutová, Ľubomíra Grešáková, Katarína Bobíková, Viera Revajová, Emília Dvorožňáková, Zuzana Ševčíková, Róbert Herich, Viera Karaffová, Rudolf Žitňan, Mikuláš Levkut

**Affiliations:** 1 Department of Morphological Disciplines, University of Veterinary Medicine and Pharmacy in Košice, Komenského 73, 041 81 Košice, Slovakia; 2 Department of Epizootiology, Parasitology and Protection of One Health, University of Veterinary Medicine and Pharmacy, Komenského 73, 041 81 Košice, Slovakia; 3 Institute of Animal Physiology Centre of Biosciences of the Slovak, Academy of Sciences, Šoltésovej 4, 040 01 Košice, Slovakia; 4 Institute of Parasitology, Slovak Academy of Sciences, Hlinkova 3, 040 01 Košice, Slovakia; 5 National Agriculture and Food Centre, Research Institute of Animal Production, Nitra, 951 41 Lužianky, Slovakia; 6 Institute of Neuroimmunology, Dúbravská cesta 9, 845 10 Bratislava, Slovakia

**Keywords:** broilers, jejunum, inorganic zinc, MUC1, MUC2, nematode

## Abstract

The effect of inorganic zinc and *Ascaridia galli* infection was studied on MUC1, MUC2 (mucin), sIgA (secretory immunoglobulin A), and metallothionein in the intestines of broilers. Thirty-five-day-old chickens (*n* = 24), COBB 500 breed, were included in a 14-day experiment. Chickens were divided into 4 groups of 6 chickens each: control ©, *Ascaridia galli* (AG), Zinc group (Zn), and combined group (AG + Zn). Samples from the intestine for determination of MUC1, MUC2, sIgA, and metallothionein were taken at 7 and 14 days during necropsy. Samples from the jejunum for determination of MUC1, MUC2, sIgA, and metallothionein were taken at 7 and 14 days during necropsy. The results demonstrated that 12 days’ administration of inorganic zinc increased production of MUC1 (*p* < 0.0001) and MUC2 (*p* < 0.001) in the *Ascaridia galli*-infected group (Ag + Zn) in comparison to control (C). The beneficial effect of zinc was also revealed in the production of sIgA (*p* < 0.0001) in the combined group (AG + Zn) at 7 days. The concentration of metallothionein increased mainly in the zinc group (*p* < 0.01) of first sampling and was upregulated in Zn and AG + Zn groups. The obtained data indicate the use of inorganic zinc as a suitable immunomodulator of intestinal immunity in *Ascaridia galli*-infected chickens.

## 1. Introduction

*Ascaridia* spp. are the most common and important worms in poultry, and infection can produce a variety of clinical signs including intestinal blockage, diarrhea, and even the death of affected avian species. The control of worms was based on the administration of benzimidazole-containing drugs [1]. Increasing resistance to the deworming drugs forced researchers to find alternative strategies for treating of parasitic infections in poultry production [2].

The life cycle is direct and birds get infected by the ingestion of embryonated eggs [3]. Ingested, embryonated eggs are transported to the duodenum and/or jejuno-ileum. In these segments of intestine, they hatch and larvae are released within 24 h [4]. Ferdushy et al. [5] showed the majority of the larvae located in the anterior half of the jejuno-ileum at 3, 5, and 7 days post-infection (dpi). The majority of larvae were found deep in the mucosa, i.e., in the lumen of the crypts during the early stage of infection [6].

The embedded larvae induced changes in the mucosal thickness such as reduced villi length at 7, 10, 14, 21, and 28 dpi, and the degree of general cellular infiltration in the lamina propria of the mucosal layer [3]. Two weeks after infection, lesions such as the formation of lymphoid aggregations and mild lymphocytic infiltration in the jejunal lamina propria of inoculated birds were seen [7].

Parasitic infections are characterized by the stimulation of immunologic mucosal mechanisms mediated by cells or antibodies [8]. Immunoglobulin A (IgA) represents the major antibody class for defense against extracellular parasites at respiratory, gastrointestinal and genitourinary mucosa [9]. The main mechanism of protection against pathogen-derived antigens provided by mucosal immunity is through IgA-producing cells.

Secretory IgA (sIgA) is released via mucosal epithelial cells and needs to be retained in the immediate mucosal environment [10]. The interaction of sIgA and mucins is very important in direct or indirect antimicrobial activity. Similarly, immunoenzymatic methods showed that IgA was the only detectable surface-bound antibody found on worms obtained from the intestine of mice of various strains throughout the course of infection [11].

Mucins provide a matrix keeping other secreted defensive molecules in a strategic location. Together these components create a slimy organized layer, which is the first barrier pathogens encounter [12]. The body’s mucosal surface is protected from pathogens, including parasites, by the gel-like extracellular matrix, mucus. The barrier function of mucins in protection of the host is well accepted as an important aspect of innate defense. It is becoming clear that mucins have a much more direct role in combating parasites and are an important part of the coordinated immune response to infection. The results of the research reveal that mucins are essential anti-parasitic effector molecules [3,13]. Intestinal mucosal tissue represents the site of infection or route of access for the majority of viruses, bacteria, yeast, protozoa, and multicellular parasites in animals. MUC1 has been demonstrated to play a dynamic role in protection of the host from infection by a wide variety of pathogens and to regulate inflammatory responses to infection [14], while MUC2 has been shown to be important in the establishment of the mucus layer [15,16].

Zinc supplementation decreases oxidative stress markers and increases phagocytosis. Moreover, Zn promotes monocyte adhesion to endothelial cells in vitro and is important for the production of pro-inflammatory cytokines [17]. Levkut et al. [16] found that both inorganic or organic zinc sources increased the expression of the MUC2 and IgA genes as well as sIgA in the intestine of broiler chickens, and finally, mucins are essential anti-parasitic effector molecules [18].

Zinc absorption occurs in the intestinal mucosa, but under normal conditions, nearly as much zinc is lost in the bile-pancreatic secretions as is absorbed by the intestine [19]. Increased feed intake of Zn supports synthesis of intracellular zinc-binding proteins, metallothioneins (MTs), isolated from different tissues primarily complexed with seven atoms of zinc per molecule of MT [20]. Metallothionein synthesis is induced by various stimuli such as metals (Cd, Pb, Zn, Cu, Hg), oxidative stress, glucocorticoids, and anticancer agents; therefore, it has been assumed that MTs play a key role in the protection against metal toxicity and oxidative stress [21].

We studied the environment in the jejunum by observation some important parameters of *A. galli* during mucosal phase after treatment with inorganic zinc. The beneficial effect of zinc on some immunological parameters in the intestine prompted us to study the effect of Zn on production of MUC1, MUC2, sIgA, and metallothionein during the mucosal phase of *Ascaridia galli*.

## 2. Materials and Methods

### 2.1. Experimental Design and Sample Collection

A total of 24 35-day-old male broiler chickens of hybrid COBB 500 were included in a 14-day experimental period. Chickens were randomly allotted to four treatment groups of 6 chickens in each: control group (C) without infection and supplementation, *Ascaridia galli*-infected group (AG), Zn-supplemented group (Zn), and combined group (AG + Zn). The infected groups (AG; AG + Zn) were inoculated orally with 500 embryonated *A. galli* eggs in 0.5 mL of phosphate buffered saline (PBS) per bird at day 2 of the experimental period, while the control birds (C) received an oral dose of 0.5 mL PBS only daily from 2 to 13 d of the experiment. Aqueous solution of inorganic form of zinc (0.5 mL/50 mg ZnSO_4_) was administered daily per os to Zn and AG + Zn groups from days 2 to 13 of the experiment.

During the whole experimental period (14 days), chickens were housed in 4 identical pens (*n* = 6 per pen). The bottom of the wire cage was littered with a hard paper, absorbent cotton, and filtering paper. All chickens were fed with commercial broiler started diet (BR1 diet) ad libitum (Table 1) and had free access to water and feed storage in common equipment for menageries (self-filling drinking bowl and feeder). Chickens were reared with a lighting regimen of 23 h light and 1 h dark. According to the requirements of the chicken’s age group, the room temperature was 18–25 °C, relative humidity ranged between 50 and 60%.

At days 7 and 14 of the experimental period, the chickens were euthanized by intraabdominal injection of xylazine (Rometar 2%, SPOFA, Praha, Czech Republic) and ketamine (Narkamon 5%, SPOFA, Praha, Czech Republic) at doses of 0.7 mL/kg body weight, and samples from the jejunum for determination of sIgA, MUC1, MUC2, and metallothionein (MT I/II) were collected during necropsy.

All procedures were carried out in accordance with the European Community guidelines (EU Directive 2010/63/EU) for the care and use of animals for scientific purposes. The specific experiment was approved by the Ethics Committee of the Veterinary Medicine and Pharmacy followed by the Committee for Animal Welfare of Ministry of Agriculture of the Slovak Republic (permit number 836/17-221).

### 2.2. Preparation of Infective Inoculum

*Ascaridia galli* eggs were obtained from adult female worms from the intestines of naturally infected chickens. The eggs were isolated from the female worm uterus according to Permin et al. [22] by a gentle mechanical maceration in 0.5 N NaOH. The eggs were embryonated in 0.1 N NaOH for 4 weeks at 28 °C in the dark. During incubation, the egg suspensions were oxygenated three times per week by stirring. Egg embryonation was evaluated microscopically on weekly basis starting from day 14. Finally, the embryonated *A. galli* eggs were stored in 0.1 N NaOH at 4–6 °C and regularly oxygenated until the infection of experimental animals. Eight-week-old egg culture was used for infection.

The infected groups were inoculated orally with 500 embryonated *A. galli* eggs in 0.5 mL of phosphate buffered saline (PBS) per bird. The infection was carried out using a plastic Pasteur pipette inserted via the esophagus to the level of the crop.

### 2.3. Intestinal Samples and Flush Protocol

The flush protocol, with some modifications, was performed as described by Holt et al. [23] and modified by Husáková et al. [24]. Tissue samples from cranial, medium, and caudal part of the jejunum (5 samples from each segment and finally added together) were excised, and 5 mol/L of warm flush solution (1 M tris/glycine buffer with 0.25% Tween 20, pH 7, Sigma Aldrich, MO, USA) was injected into the intestinal lumen with a 10-cc syringe and an 18-ga needle. The solution was aspirated and injected into the intestine several times to flush the secretions from the intestine wall. Then, the cecum contents were collected into the syringe and dispelled into a tube. The intestine flushes were centrifuged for 5 min at 12,000 g (Hettich Rotina 420R Centrifuge, DJB Labcare, Newport Pagnell, UK), and the supernatants from each sample were frozen at −20 °C until the enzyme-linked immunosorbent assay (ELISA) procedure was done.

### 2.4. Enzyme-Linked Immunosorbent Assay (ELISA) sIgA

Total IgA was detected by using chicken IgA ELISA kit (Kamiya Biomedical Company, Tukwila, WA, USA). Briefly, test samples, diluted 1:5 in 1× diluent solution, were added at 100 μL per well in duplicate wells. Undiluted positive and negative controls were also applied at 100 μL per well in duplicate wells. The plate was incubated at room temperature for 20 min, followed by three washings with wash solution. Anti-chicken HRP conjugate was added at 100 μL per well, followed by incubation at room temperature for 20 min, and three washings in wash buffer. One hundred microliters of 3,3,5,5-tetramethyl benzidine substrate solution was added into each well, and incubated at room temperature for 15 min, followed by addition of stop solution at 100 μL per well. Absorbance at 450 nm (A (450)) was measured in a Microplate reader (Revelation Quicklink, Opsys MR, Dynex Technologies, Chantilly, VA, USA). Interpretation was carried out using a calibration curve prepared according to the manufacturer’s protocol.

### 2.5. Enzyme-Linked Immunosorbent Assay (ELISA) of MUC-1 and MUC-2

For detection of total MUC1 and MUC2, we used a chicken MUC-1 ELISA kit and a chicken MUC-2 ELISA kit (both from Kamiya Biomedical Company, Tukwila, WA, USA). The procedure was the same for both mucin-1 and mucin-2. Ninety-six-well microtiter plates were coated with affinity purified anti-chicken MUC antibody. After incubation, each plate was washed, and 50 μL of substrate solution was added into each well. The samples were diluted 1:5 with PBS (pH 7.0–7.2) and added in 100 μL doses into pre-designated wells in duplicates. Then, 10 μL of balance solution and 50 μL of conjugate binding with horseradish peroxidase in stabilizing buffer was applied into the plate wells and incubated at 37 °C for 1 h. The reaction was stopped with 50 μL of stop solution, the plates were incubated at 37 °C for 10 to 15 min, and then absorbance was measured spectroscopically at 450 nm on a microplate reader. Interpretation was done using a calibration curve prepared according to the manufacturer’s protocol.

### 2.6. Enzyme-Linked Immunosorbent Assay (ELISA) of Metallothionein (MT I/II)

Metallothionein I/II was detected by using an anti-MT ELISA kit (Kamiya Biomedical Company, Tukwila, WA, USA). Briefly, assay samples (50 µL) were added into each well. Then, anti-MT antibody (50 µL) was added to the same wells. The plate was incubated at 4 °C. After incubation, the wells were washed 3 times with buffer solution. Anti-rabbit HRP antibody was added to each well and incubated for 1 h at room temperature. After washing of wells, 100 µL of 3.3.5.5-tetramethyl benzidine (TMB) substrate solution was added into each well, and incubated for 10 min at room temperature, followed addition of stop solution. Absorbance at 450 nm (A (450)) was measured in a Microplate reader (Revelation Quicklink, Opsys MR, Dynex Technologies, Chantilly, VA, USA). Interpretation was carried out using calibration curve prepared according to the manufacturer’s protocol.

### 2.7. Histological Procedure

Jejunal samples were fixed in 10% neutral buffered formalin and prepared using routine paraffin embedding techniques. Three consecutive sections (5 µm) from each jejunum were stained using hematoxylin and eosin and observed by light microscopy (Nikon, Tokyo, Japan). The photos were prepared by camera in the NIS-Elements Advanced Research Programme (Ver. 3.00, Nikon, Tokyo, Japan).

### 2.8. Statistical Analysis

Statistical analysis of data was performed using two-way ANOVA with Tukey post hoc analysis using the statistical program GraphPad PRISM version 6.00. Differences between the mean values for different treated groups were considered statistically significant at *p* < 0.05, *p* < 0.01, *p* < 0.001, *p* < 0.0001. Values in figures are given as standard error of the mean (±SEM).

## 3. Results

### 3.1. Clinical Sings, Gross Lesions, Histological Changes

**Figure 1:**
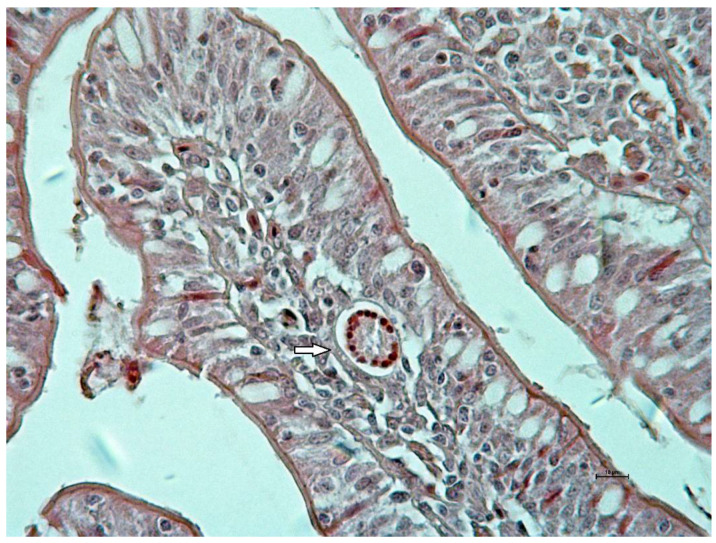
Cross section of *Ascaridia galli* larva (arrow) in edematic and inflamed jejunal lamina propria mucosae at 7 days post infection in AG group (Hematoxylin-Eosin staining, bar 10 μm).

Clinical examination did not show signs in relation to *A. galli* infection during the experiment. Macroscopically, small intestine mucosa demonstrated focal hemorrhages located mainly on jejunal mucous membrane at days 7 and 14 of the experiment. Histologically, larvae were seen mainly in lamina propria of tunica mucosae. Microscopic examination of mucosa revealed edema and cellular infiltration presented by lymphoid cells mixed with eosinophils during both samplings (Figure 1). In the mucosal phase, ten larvae were calculated. Seven were located in lamina propria mucosae and three were found in the lumen of crypts.

### 3.2. Evaluation of MUC1 and MUC2

**Figure 2:**
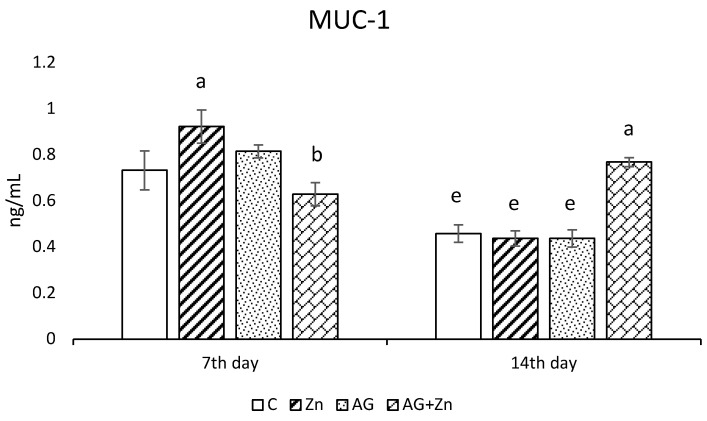

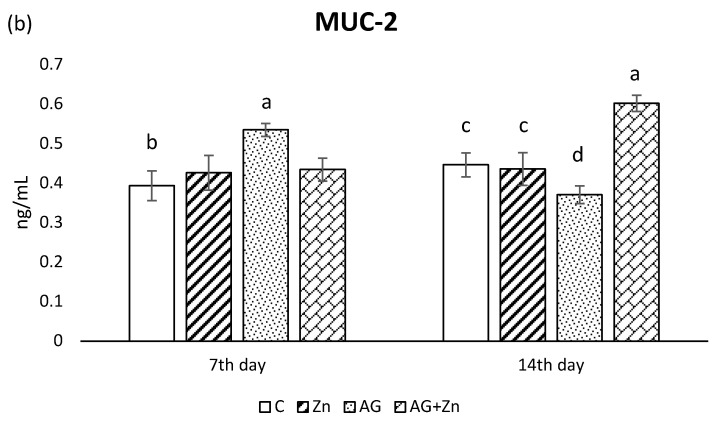
Effect of oral zinc administration on mucin production (**a**) MUC1, and (**b**) MUC2 in the jejunum of control and infected chickens by *A. galli* on day 7 and 14 post infection. Means with different superscripts are significantly different (a,b *p* < 0.05; a,c *p* < 0.01; a,d *p* < 0.001; a,e *p* < 0.0001).

Zinc administration increased production of MUC1 (Figure 2a) significantly (*p* < 0.05) compared to infected chickens (AG + Zn) on day 7, while MUC1 production was increased in AG + Zn group to others (*p* < 0.0001) on day 14 of the study.

The *A. galli* group (AG) after 7 days infection showed in jejunal segments the highest MUC2 production (Figure 2b) compared to non-infected and infected chickens given Zn (Zn and AG + Zn groups) without significance, as well to controls (C group) with statistical difference on the level *p* < 0.05. On day 14 of the study, MUC2 production increased in the group AG + Zn (*p* < 0.01) to C and Zn, and (*p* < 0.001) to AG groups.

### 3.3. Determination of sIgA

**Figure 3:**
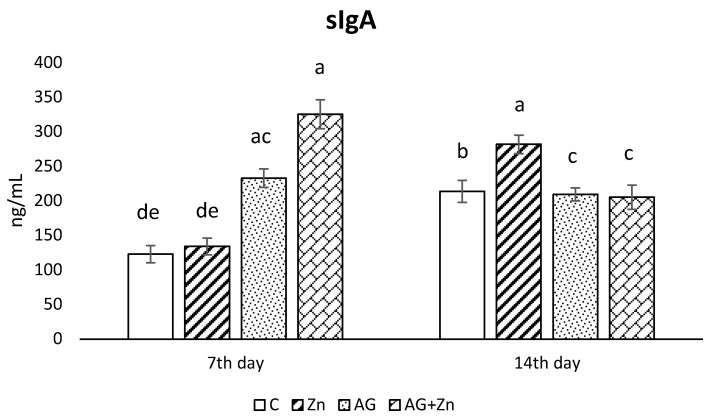
Effect of oral zinc administration on mucosal production of sIgA in the jejunum of control and infected chickens by *A. galli* on day 7 and 14 post infection. Means with different superscripts are significantly different (a,b *p* < 0.05; a,c *p* < 0.01; a,d *p* < 0.001; a,e *p* < 0.0001).

Production of sIgA in the jejunum (Figure 3) rapidly increased in both infected groups: AG to control chickens (C, Zn *p* < 0.001) on day 7, with the highest sIgA production in AG + Zn group (C, Zn *p* < 0.0001, Ag *p* < 0.01). On day 14 of the study, the increase of sIgA was recorded in Zn group comparing to control group (*p* < 0.05), and both infected groups by *A. galli* (*p* < 0.01).

### 3.4. Measurement of Metallothionein

**Figure 4:**
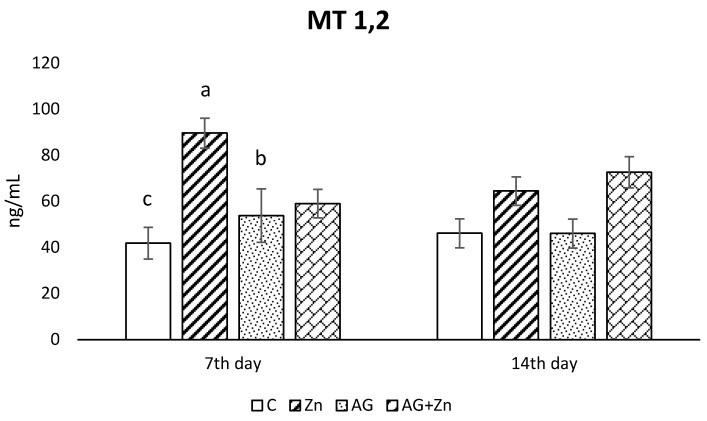
Effect of oral zinc administration on concentration of metallothioneins 1, 2 in the jejunum of control and infected chickens by *A. galli* on day 7 and 14 post infection. Means with different superscripts are significantly different (a,b *p* < 0.05; a,c *p* < 0.01).

Zinc administration caused the increase of metallothionein concentration (Figure 4) in the jejunum of uninfected chickens compare to control group without Zn (*p* < 0.01) and AG group (*p* < 0.05) on day 7 of the study. An insignificant increase of metallothionein concentration was recorded in both groups given Zn (Zn, AG + Zn) compared to chickens without Zn on day 14 post infection.

## 4. Discussion

The normal habitat of the parasitic stages of *Ascaridia galli* is the small intestine and preferentially resides in the jejunum; therefore, the effect of zinc was concentrated at the lesions induced by the parasite in the jejunum in our experiment [3,6]. Histopathologically, the majority of *A. galli* larvae were located in the lamina propria of the tunica mucosae on day 7 and 14 of the infection. Some authors mentioned that the duration of the mucosal phase can be from 3 to 54 days [25], while others observed larvae in the mucosa only for several days [26]. The duration of the mucosal phase could depend on storage and incubation conditions. In our trial, the embryonated eggs were stored at 4 °C to 6 °C.

The primary role of the mucus barrier is to act as a physical barrier to protect the underlying intestinal epithelium. In the chicken gastrointestinal tract, mucin-1 (MUC1) is cell surface mucin and to access its receptor on the host cell, invading pathogens must first circumvent the protective mucus layer, which is composed of a network of mucins [14]. MUC2 is included into gel-forming mucins containing proteins with a structural, antimicrobial, and regulatory function which have been identified to be present during parasitic infection [27,28]. Increased jejunal production of MUC2 in infected chickens on day 7 in our study indicates the subvertation of the mucus barrier and allows the parasite to establish within their intestinal niche. Production of MUC2 was significantly upregulated during *Trichinella spiralis* infection in mice [29], and experimentally infected pigs by *Trichinella spiralis* had increased mucins stored in goblet cells [30]. Ablation of MUC2 in vivo led to delayed expulsion of acute *T. muris* infection, even though a Th2-mediated immune response prevailed [13]. Moreover, significant upregulation of mucin production was confirmed in porcine model of Trichuris suis infection [31]. *Trichuris muris* infection caused increased levels of MUC2 transcripts only at the site of the parasite colonization (mouse caecum) at the time of worm expulsion [13].

During gastrointestinal nematode infection, there is a change in the mucus barrier composition and properties, changes in mucin expression, and glycosylation parasitic invasion [32]. On the other hand, longer per os administration of zinc increased MUC2 and MUC1 production in infected chickens by *A. galli* in our study. Similarly, Levkut at al. [16] indicated that feed supplementation with either the inorganic or organic zinc sources led to increased expression of the MUC2 in small intestine of chickens. Since the mucus barrier can actively lead to deleterious effects on parasite viability [32], our results suggest the important role of Zn in the increased quantity of mucins in the chicken digestive tract. The administration of zinc could intensify the protective role of MUC2 in infected animals.

Production of sIgA in the jejunum of *Ascaridia galli*-infected chickens demonstrates increased antibody reaction to infected parasite. These data were consistent with the upregulation of IgA gene in infected chicken by *A. galli* given Zn on day 7 [33]. Administration of Zn for a 14-day period revealed increase of IgA production in the jejunum of intact chickens. Similar results were recorded in chickens fed the supplemented diets by Zn, with increased level of sIgA in the intestinal flush [16].

Intestinal metallothioneins (MTs) maintain Zn homeostasis by regulation of Zn absorption and by intracellular Zn-binding capacity of MTs in enterocytes [34]. In addition, MTs as free radical scavengers play an important role in the antioxidant defense system of cells [35]. A 7-day oral administration of Zn in our study increased concentration of MT 1 and 2 in the jejunum of intact chickens, but longer Zn intake elevated MT concentrations in intact as well as in infected chickens. Our results indicate that long-term intake of Zn can protect the mucosa of small intestine against oxidative stress caused by *A. galli* infection due to the increased MT concentrations and can help to regenerate intestinal mucosa. It is known that in the presence of zinc, MT is quickly reconstituted [36].

## 5. Conclusions

Our results demonstrated that 12-day administration of inorganic zinc increased production of MUC1 and MUC2 in *Ascaridia galli*-infected groups when compared to controls. The beneficial effect of zinc on the infected group was also revealed in the higher production of sIgA at 7 days. The concentration of metallothionein of the pure zinc group was significantly increased in first sampling and insignificantly in both zinc groups (Zn, AG + Zn) at 14 days. A better understanding of the changes in mucins’ composition could highlight the approach to the clearance of parasites during infection. In the future, the beneficial effect of zinc on *Ascaridia galli*-infected chickens is necessary to evaluate in relation to the viability of the embryonated eggs in mucus and their expulsion.

## Figures and Tables

**Table 1:** Composition of commercial diet BR1.

Ingredients g/kg	BR1
Wheat	290
Maize	300
Soybean meal	320
Rapeseed oil	40
Fish meal	20
Limestone	12
Dicalcium phosphate	10
Sodium chloride	2
DL-methionine	1
Vitamin-mineral mix	5
**Composition by analysis (g/kg)**	
Dry matter	899.9
Crude protein	232.7
Fat	64.5
Dietary fiber	22.7
Ash	53
Ca	90.4
P total	69.6
Metabolize energy	11.515

## Data Availability

Data are available upon request from the authors.

## References

[1] Anand K., Wakode S. (2017). Development of drugs based on benzimidazole heterocycle: Recent advancement and insights. Int. J. Chem. Stud..

[2] Collins B., Jordan B., Vidvashnkar A.N., Castro P.J., Fowler J., Kaplan R.M. (2021). Impact of fenbendazole resistance in *Ascaridia dissimilis* on the economic production in turkeys. Poult. Sci..

[3] Luna-Olivares L.A., Kyvsgaard N.C., Ferdushy T., Nejsum P., Thamsborg S.M., Roepstorff A., Iburg T.M. (2015). The jejunal cellular responses in chickens infected with a single dose of *Ascaridia galli* eggs. Parasitol. Res..

[4] Hansen M., Terhaar C., Turner D. (1956). Importance of the egg shell of *Ascaridia galli* to the infectivity of its larva. J. Parasitol..

[5] Ferdushy T., Luna-Olivares L.A., Nejsum P., Roepstorff A., Thamsborg S.M., Kyvsgaard N.C. (2013). Population dynamics of *Ascaridia galli* following single infection in young chickens. Parasitology.

[6] Luna-Olivares L.A., Ferdushy T., Kyvsgaard N.C., Nejsum P., Thamsborg S.M., Roepstorff A., Iburg T.M. (2012). Localization of *Ascaridia galli* larvae in the jejunum of chickens 3 days post infection. Vet. Parasitol..

[7] Schwarz A., Gauly M., Abel H., Daş G., Humburg J., Rohn K., Breves G., Rautenschlein S. (2011). Immunopathogenesis of *Ascaridia galli* infection in layer chicken. Dev. Comp. Immunol..

[8] Else K.J. (2005). How gastrointestinal nematodes outwitted the immune system?. Parasite Immunol..

[9] Antonella B., Politis I., Pecorini C., Fusi E., Chronopoulou R., Dell’orto V. (2005). Biological effects of milk proteins and their peptides with emphasis on that related gastrointestinal ecosystem. J. Dairy Res..

[10] Herich R. (2016). Is the role of IgA in local immunity completely known?. Food Agric. Immun..

[11] Matsuzawa K., Abe M., Shirakura T., Zhao W., Nakamura F. (2008). Spontaneous worm expulsion and intestinal IgA response in mice infected by *Vampirolepis nana*. Parasitol. Int..

[12] Linden S.K., Karlsson N.G., Korolik V., McGuckin M.A. (2008). Mucins in the mucosal barrier to infection. Mucosal Immunol..

[13] Hasnain S.Z., Wang H., Ghia J.E., Hag N., Deng Y., Velcich A., Grencis R.K., Thornton D.J., Khan W.I. (2010). Mucin gene deficiency in mice impairs host resistance to an enteric parasitic infection. Gastroenterology.

[14] Dhar P., McAuley J. (2019). The role of the cell surface mucin MUC-1 as a barrier to infection and regulator of inflammation. Front. Cell Infect. Microbiol..

[15] Zhang Q., Eicher S.D., Applegate T.J. (2015). Development of intestinal mucin 2, IgA, and polymeric Ig receptor expressions in broiler chickens and Pekin ducks. Poult. Sci..

[16] Levkut M., Husáková E., Bobíková K., Karaffová V., Levkutová M., Ivanišinová O., Grešáková Ľ., Čobánová K., Reiterová K., Levkut M. (2017). Inorganic or organic zinc and MUC-2, IgA, IL-17, TGF-β gene expression and sIgA secretion in broiler chickens. Food Agric. Immunol..

[17] Bonaventura P., Benedetti G., Albarède F., Miossec P. (2015). Zinc and its role in immunity and inflammation. Autoimmun. Rev..

[18] Hasnain S.Z., Gallagher A.L., Grencis R.K., Thorton D.J. (2013). A new role for mucins in immunity: Insights from gastrointestinal nematode infection. Int. J. Biochem. Cell Biol..

[19] Walsh C.T., Sandstead H.H., Prasad A.S., Newberne P.M., Fraker P.J. (1994). Zinc: Health effects and research priorities for the 1990s. Environ. Health Perspect..

[20] Andrews G.K. (1990). Regulation of metallothionein gene expression. Prog. Food Nutr. Sci..

[21] Sato M., Kondoh M. (2002). Recent studies on metallothionein: Protection against toxicity of heavy metals and oxygen free radicals. Tohoku J. Exp. Med..

[22] Permin A., Pearman M., Nansen P., Bisgaard M., Frandsen F. (1997). An investigation on different media for embryonation of *Ascaridia galli* eggs. Helminthologia.

[23] Holt P.S., Gast R.K., Porter R.E., Stone H.D. (1999). Hyporesponsiveness of the systemic and mucosal humoral immune systems in chickens infected with *Salmonella* enterica serovar Enteritidis at one day of age. Poult. Sci..

[24] Husáková E., Bobíková K., Stašová D. (2015). Total IgA in spleen, bursa and intestine of chickens pretreated with *E. faecium* AL41 and challenged with *Salmonella* Enteritidis PT4. Food Agric. Immunol..

[25] Permin A., Hansen J.W. (1998). Epidemiology, diagnosis and control of poultry parasite. FAO Animal Health Manual.

[26] Herd R.P., McNaught D.J. (1975). Arrested development and the histotrophic phase of *Ascaridia galli* in the chickens. Int. J. Parasitol..

[27] Dehlawi M.S., Mahida Y.R., Hughes K., Wakelin D. (2006). Effect of *Trichinella spiralis* infection on intestinal pathology in mice lacking interleuki-4 (IL-4) or intestinal trefoil factor (ITF/TFF3). Parasitol. Int..

[28] Herbert B.R., Yang J.O., Hogan S.P., Groswitz K., Khodoun M., Munitz A., Orekov T., Perkins C.H., Wang Q., Brombacher F. (2009). Intestinal epithelial cell secretion of RELM-βprotect against gastrointestinal worm infection. J. Exp. Med..

[29] Shekels L.L., Anway R.E., Lin J., Kennedy M.W., Garside P., Lawrence C.F., Ho S.B. (2001). Coordinadted Muc2 and Muc3 mucin gene expression in *Trichinella spiralis* infection in wild-type and cytokine-deficient mice. Dig. Dis. Sci..

[30] Theoropoulos G., Hicks S.J., Corfield A.P., Miller B.G., Kapel C.M.O., Trivizaki M., Balaskas C., Petrakos G., Carrington S.D. (2005). *Trichinella spiralis*: Enteric mucin-related response to experimental infection in conventional and SPF pigs. Exp. Par..

[31] Thomsen L.E., Knudsen K.E.B., Hedernann M.S., Roepstorff A. (2006). The effect of dietary carbohydrates and *Trichuris suis* infection on pig large intestine tissue structure, epithelial cell proliferation and mucin characteristics. Vet. Parasitol..

[32] Sharpe C., Thornton D.J., Grencis R.K. (2018). A sticky end for gastrointestinal helminths; the role of the mucus barrier. Parasite Immunol..

[33] Karaffová V., Revajová V., Dvorožňaková E., Grešáková Ľ., Levkut M., Ševčíková Z., Herich R., Levkut M. (2021). Effect of inorganic zinc on selected immune parameters in chicken blood and jejunum after *A. galli* infection. Agriculture.

[34] Ruttkay-Nedecky B., Nejdl L., Gumulec J., Zitka O., Masarik M., Eckschlager T., Stiborova M., Adam V., Kizek R. (2013). The role of metallothionein in oxidative stress. Int. J. Mol. Sci..

[35] Kimura T., Kambe T. (2016). The functions of metallothionein and ZIP and ZnT transporters: An overview and perspective. Int. J. Mol. Sci..

[36] Kang Y.J. (2006). Metallothionein redoc cycle and function. Exp. Biol. Med..

